# Assessment of cutaneous axon-reflex responses to evaluate functional integrity of autonomic small nerve fibers

**DOI:** 10.1007/s10072-020-04293-w

**Published:** 2020-03-03

**Authors:** Mido M. Hijazi, Sylvia J. Buchmann, Annahita Sedghi, Ben M. Illigens, Heinz Reichmann, Gabriele Schackert, Timo Siepmann

**Affiliations:** 1 Department of Neurosurgery, University Hospital Carl Gustav Carus, Technische Universität Dresden, Fetscherstr 74, 01307 Dresden, Germany; 2 Department of Anaesthesiology, Operative Intensive Care Medicine and Pain Medicine, Vivantes Klinikum Spandau, Berlin, Germany; 3 Department of Neurology, University Hospital Carl Gustav Carus, Technische Universität Dresden, Dresden, Germany; 4 Department of Neurology, Beth Israel Deaconess Medical Center, Harvard Medical School, Boston, MA USA

**Keywords:** Vasomotor, Sudomotor, Pilomotor, Axon-reflex, Autonomic

## Abstract

Cutaneous autonomic small nerve fibers encompass unmyelinated C-fibers and thinly myelinated Aδ-fibers, which innervate dermal vessels (vasomotor fibers), sweat glands (sudomotor fibers), and hair follicles (pilomotor fibers). Analysis of their integrity can capture early pathology in autonomic neuropathies such as diabetic autonomic neuropathy or peripheral nerve inflammation due to infectious and autoimmune diseases. Furthermore, intraneural deposition of alpha-synuclein in synucleinopathies such as Parkinson’s disease can lead to small fiber damage. Research indicated that detection and quantitative analysis of small fiber pathology might facilitate early diagnosis and initiation of treatment. While autonomic neuropathies show substantial etiopathogenetic heterogeneity, they have in common impaired functional integrity of small nerve fibers. This impairment can be evaluated by quantitative analysis of axonal responses to iontophoretic application of adrenergic or cholinergic agonists to the skin. The axon-reflex can be elicited in cholinergic sudomotor fibers to induce sweating and in cholinergic vasomotor fibers to induce vasodilation. Currently, only few techniques are available to quantify axon-reflex responses, the majority of which is limited by technical demands or lack of validated analysis protocols. Function of vasomotor small fibers can be analyzed using laser Doppler flowmetry, laser Doppler imaging, and laser speckle contrast imaging. Sudomotor function can be assessed using quantitative sudomotor axon-reflex test, silicone imprints, and quantitative direct and indirect testing of sudomotor function. More recent advancements include analysis of piloerection (goose bumps) following stimulation of adrenergic small fibers using pilomotor axon-reflex test. We provide a review of the current literature on axon-reflex tests in cutaneous autonomic small fibers.

## Introduction

Autonomic small fibers mediate neural control of vital organs such as the cardiovascular, bronchopulmonary, gastrointestinal, and urogenital system as well as the skin. Autonomic neuropathies are defined by damage to lightly myelinated A-delta and unmyelinated C-fibers. These “small fibers” constitute the most peripheral segments of the sympathetic and parasympathetic branch of the autonomic nervous system. Autonomic neuropathies comprise a group of etiopathogenetically heterogenous disorders, which can manifest with vegetative symptoms such as orthostatic hypotension, reduced heart rate variability, sexual and urogenital dysfunction, and dyshidrosis of the skin, frequently reducing quality of life [1, 2]. Autonomic neuropathies are therefore of high clinical relevance. This is further highlighted by an increase of mortality in those autonomic neuropathies that impair neural control of the cardiovascular system [3]. Diabetes is the most prevalent aetiology of autonomic peripheral neuropathy. Further common causes include amyloidosis, synucleinopathies such as Parkinson’s disease, and autoimmune diseases such as acute inflammatory demyelinating polyneuropathy. Moreover, autonomic neuropathy can be associated with infectious diseases such as acquired immune deficiency syndrome, hereditary disorders such as Fabry disease, channelopathies, hereditary sensory, and autonomic neuropathies as well as neurotoxins such as Shiga toxin or cisplatin [4]. Aetiologies can be classified into seven groups as shown in Table 1.

**Table 1:** Causes of autonomic small-fiber neuropathies

Endocrine	Hereditary	Amyloidotic	Toxic	Infectious	Autoimmune and paraneoplastic
Diabetes mellitus type 1	Hereditary sensory and autonomic neuropathy type I (HSAN I)	AA amyloidosis (acute phase proteins)	Acrylamide (substrate of polymer production)	Human immunodeficiency virus disease	Guillain-Barre syndrome
Diabetes mellitus type 2	Hereditary sensory and autonomic neuropathy type II (HSAN II)	AL amyloidosis (light chains)	Vacor (N-3-pyridylmethyl- N′-p-nitrophenyl urea)	Chagas disease	Axonal variants of Guillain-Barré syndrome
	Hereditary sensory and autonomic neuropathy type III (HSAN III)	AE-amyloidosis (protein hormones)	Vincristine (chemotherapeutic agent)	Leprosy (Mycobacterium leprae)	Chronic inflammatory demyelinating polyradiculoneuropathy
	Hereditary sensory and autonomic neuropathy type IV (HSAN IV)	AP amyloidosis (prealbumin)	Cisplatin (chemotherapeutic agent)	Diphtheria (Corynebacterium diphtheriae)	Chronic inflammatory axonal polyradiculoneuropathy
	Hereditary sensory and autonomic neuropathy type V (HSAN V)	AB amyloidosis (β2 microglobulin	Paclitaxel (chemotherapeutic agent)		Acute autoimmune autonomic neuropathy
	Triple-A syndrome	amyloidosis (various proteins)	Amiodarone (antiarrhythmic)		Rheumatoid and systemic syndromes with Ro, −La and/or M3 antibodies
	Tangier disease	ATTR-amyloidosis (Transthyretin)	Pentamidine (antiprotozoan agent)		Paraneoplastic syndromes with Hu, −PCA-2, −CRMP-5 and/or amphiphysin antibodies
	Fabry disease		Perhexelin (antipectan gum carnitine palmitic transferase inhibitor)		
	Multiple endocrine neoplasia 2b (MEN)		Botulinum (botulism)		
	Channelopathies		Ciguatoxin and maitotoxin (Ciguatera)		

A table of disorders leading to damage to autonomic small fibers. Each column represents a disease category [1, 2, 4]

In order to improve detection of autonomic neuropathies, research has focused on the development of techniques to infer integrity of the peripheral autonomic nervous system. Cutaneous small fibers have been identified as diagnostic target because of their easy anatomic accessibility and early pathophysiological involvement in highly prevalent disorders such as Parkinson’s disease and diabetes. Autonomic small fibers of the skin comprise sudomotor fibers innervating sweat glands, vasomotor fibers innervating arterial blood vessels, and pilomotor fibers innervating pilomotor muscles attached to hair follicles (Mm. arrectores pilorum).

Research has recently focused on evaluating small fiber function in patients with synucleinopathies such as Parkinson’s disease, as in these patients, cutaneous small fibers can be damaged by intraneural deposition of alpha-synuclein in even in the prodromal and early disease stages [5–7]. Therefore, techniques to assess small fiber function might allow early diagnosis and initiation of treatment. Microneurography is a minimally invasive technique to assess small nerve fiber integrity, which is mainly used in research studies and experimental settings. It is not widely used for clinical diagnostic purposes as it is technically demanding and requires extensive training. Immunohistochemical analysis of invasive skin punch biopsies allows assessment of small nerve fiber integrity on a structural level.

Axon-reflex-based assessment of vasomotor, sudomotor, and pilomotor small fibers may supplement these techniques and provide non-invasive tools to detect and monitor small fiber neuropathy [8–11]. The physiological concept of the axon-reflex was introduced by Langley in the 1890s [12, 13]. Some authors also refer to earlier investigations of the axon-reflex by Sokovnin and Rozhanskiy [14]. This reflex evokes a localized response only in those cells that are innervated by the neuron the action potential was induced in. This neurophysiological pathway differs from spinal cord reflexes as the reflex arc does neither include the central nervous system nor any synaptic connections to other neurons. This observation gained more attention in the 1940s after Sir Thomas Lewis has suggested that the axon-reflex in cutaneous vasomotor nerve fibers is responsible for vasodilation in adjacent skin areas after scratching [15]. Since then, axon-reflex-mediated responses in vasomotor and sudomotor (and more recently in pilomotor) have been subject to multiple research studies.

The axon-reflex can be induced by stimulation of small nerve fiber terminals in the skin. An action potential is generated which is then conducted to an upstream axon branching point where the potential switches to neighboring small fibers to travel backward. Thus, the potential reaches the distal fiber endings in a skin region adjacent to the area of stimulation (“indirect area”). Thereby, a local response of the innervated organ is elicited, i.e., dilation of arterial skin vessels, sweating, or piloerection [16–18]. The axon-reflex can be elicited by iontophoresis of cholinergic agents in vasomotor or sudomotor fibers, adrenergic agents in pilomotor fibers, respectively, as well as by thermal, mechanic, or electric stimuli. The response is assessed quantitatively in the indirect skin area surrounding the “direct area” where the stimulus is applied.

We aimed to provide a narrative review of the current literature on axon-reflex assessment. Moreover, we sought to discuss the drawbacks and potential future implications of these tests.

## Assessment of vasomotor function

**Fig. 1:**
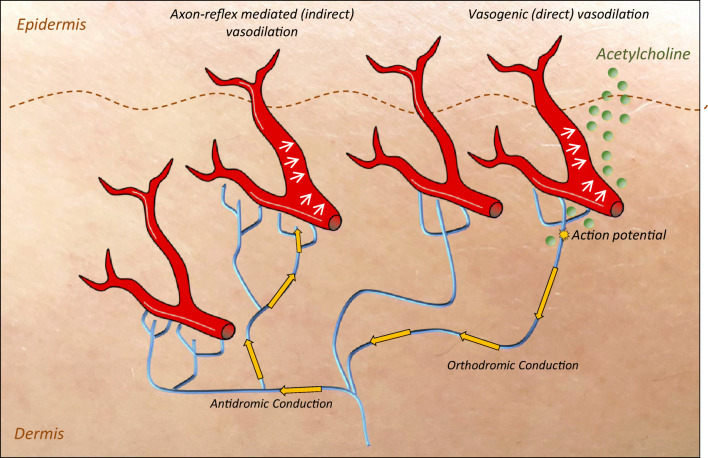
The vasomotor axon-reflex. Epidermal noceptive C-fibers are stimulated via iontophoresis of acetylcholine, which induces local vasodilation of small blood vessels (direct area). Neurophysiologically seen acetylcholine generates an action potential in the unmyelinated C-fiber, which is orthodromically conducted toward the spinal cord. At branching points of the epidermal nerve fibers, the action potential is antidromically conducted to neighboring nerve endings, inducing a release of vasoactive substances with consecutive vasodilation in this neighboring area. This local spread of action potential is known as axon-reflex and the unstimulated skin area is called indirect area

The cutaneous microcirculation contributes substantially to thermal haemostasis of the human body and is anatomically based on two horizontal plexuses located in the dermis. Neural control of microvascular reactivity in these plexuses is mediated by vasomotor autonomic small nerve fibers [19]. In these fibers, the axon-reflex can be evoked directly by pharmacological, thermal, electrical, or mechanical stimuli. Topical stimulation of terminal nerve endings of the cutaneous small fibers with acetylcholonine (Ach) induces an action potential in unmyelinated C-fibers. This potential is then orthodromically conducted to an axon branch point, where it is redirected to a group of neighboring axons to antidromically reach neighboring nerve terminals of C-fibers innervating the blood vessels. Consecutively, vasoactive substances, such as substance P (SP) and calcitonin gene-related peptide (CGRP), are released from C-fiber nerve terminals causing a vasodilatory response adjacent to the skin area of direct Ach stimulation [9, 20] (Fig. 1). The vasodilation caused by this mechanism can be quantified as a measure of neurogenic blood flow. Agents such as Ach also act directly on the endothelium, causing a vasogenic blood flow response. Vasogenic and neurogenic responses can be differentiated topographically. While vasogenic blood flow responses occur in the skin area of stimulation (e.g., iontophoretic application of Ach), neurogenic axon-reflex-mediated blood flow responses are evoked in a skin region adjacent to the area of stimulation.

Laser Doppler-based techniques such as laser Doppler flowmetry (LDF), laser Doppler imaging (LDI), and laser speckle contrast imaging (LSCI) are used to quantify axon-reflex-mediated vasodilation as a measure of vasomotor small fiber function. They provide an index of skin perfusion by measuring the Doppler shift induced by coherent monochromatic light scattering by moving red blood cells. The received signal is quantified as the product of average red blood cell velocity and concentration. This signal is often referred to as flux because it does not provide an exact measure of flow (ml/min) [21]. Limitations of laser Doppler techniques comprise inconsistencies in the standardization of settings used for induction of the axon-reflex such as density and Ach concentrations. Further limitations include technical demands as well as intra-and interindividual variability. Moreover, technical susceptibility to environmental factors such as light, temperature, hairs, and humidity may explain why laser Doppler assessment is predominantly provided in specialized autonomic centers.

## Laser Doppler flowmetry

### Procedure

Laser Doppler flowmetry (LDF) is used to assess axon-reflex-mediated blood flow responses to Ach. Iontophoresis of 10% Ach solution stimulates the endothelium-dependent production of SP, CGRP, and nitric oxide (NO) leading to microvascular vasodilation. The intensity of the detected blood flow in the indirect area of response constitutes a measure of vasomotor small fiber function [20, 22]. In the LDF technique, the laser beam is directed towards a single predefined spot on the skin in a pre-set distance from the iontophoretic application point. The received backscattered signal from moving blood cells is quantified as the product of average red blood cell velocity and concentration (flux) [21]. A linear relationship between flux and actual flow has been demonstrated [22]. The technique quantifies the vasodilator response following Ach iontophoresis at a single predefined skin point with temporal resolution in a predefined distance from the iontophoresis capsule [23]. Single-point LDF with one transmitting and one receiving optical fiber on a fixed area of the skin is accurate at quantifying fast changes in blood flow. Blood flow is measured over a small volume (1 mm or smaller) with a high sampling frequency (often 32 Hz) [24]. As opposed to single point assessment of blood flow, integrating probes, which are made of seven to eight optical fiber pairs, assess blood flow in multiple points, thereby decreasing spatial variability and improving reproducibility by averaging the signal from different scattering volumes [25, 26].

### Limitations

The regional heterogeneity of skin perfusion due to intra- and inter-individual differences of skin anatomy is associated with high spatial variability, reducing the interpretability and reproducibility of measurements, particularly when single point LDF probes are used [24]. While LDF can capture intensity of changes in blood flow at predefined points, it fails to assess the spatial spread of axon-reflex-mediated increase in blood flow.

### Perspective

LDF enables the detection of cutaneous perfusion differences between groups of patients with peripheral neuropathy and healthy individuals but may not reach enough sensitivity to compare single individuals to normative datasets, thereby limiting its clinical utility [1]. Although LDF cannot be used to screen patients for neuropathy, the technique may provide useful supplementary information particularly in longitudinal intra-patient disease monitoring or in cases of extreme vasomotor impairment [27]. In the clinical setting, results on small fiber function obtained with LDF might be valuable when showing consistency with other assessment techniques with lower variability. In the scientific setting, LDF has been used to study familial dysautonomia and has helped to understand the relationship between receptors and innervation in autonomic dysfunction [28]. Furthermore, research using LDF in patients with Huntington’s disease displaying vasomotor impairment was able to lend evidence to the hypothesized involvement of the autonomic nervous system in the disease’s pathophysiology [29].

## Laser Doppler imaging

### Procedure

Like in LDF, blood flow is quantified following induction of axon-reflex-mediated vasodilation by iontophoresis of Ach into the upper dermal layers. The technique, however, differs from the single point technique LDF by allowing for spatial assessment of the predefined skin area which is scanned by a moving laser beam to generate a two-dimensional perfusion map. The imager instrument is installed in a static position and therefore has a defined distance from the scanned skin area [30, 31]. Repeated scanning allows for evaluation of the response with temporal resolution. The two-dimensional perfusion map perfusion map thus changes over time to display changes in blood flow. The map itself is generated from recorded perfusion images of single cells (perfusion units). Therefore, perfusion values of every single cell measured in the predefined skin area are recorded, and once single cell perfusion increases above a predefined threshold, the color of the respective cell changes. Thresholds used for color coding vary among LDI analysis techniques.

#### Flare area method

The flare area is defined area of increased blood flow in the indirect region of axon-reflex-mediated increased blood flow. In order to define the flare area, the number of perfusion units exceeding a certain perfusion threshold prior to intophoresis is subtracted from the number of perfusion units exceeding the very same perfusion threshold after iontophresis of Ach. The perfusion threshold itself is defined as 125 perfusion units. This fixed setting is based on a previous study using a capsaicin-based human model of small fiber neuropathy where a threshold of 125 PU displayed the largest difference in flare area between pre-and post-iontophoretic blood flow among subjects [31].

#### Baseline perfusion method

In the baseline perfusion method, the blood flow threshold is calculated as two standard deviations of the measured baseline perfusion of the skin surface. Consequently, the area of axon-reflex-mediated increased blood flow is defined as the area of all cells above this threshold [32].

#### Resting blood flow method

The axon-reflex-mediated spread of increased blood flow is assigned to the area in which a three-fold increase of the baseline blood flow is registered within the predetermined skin surface. The method was designed using an arbitrarily chosen threshold intending to ensure detection of meaningful differences [33].

### Limitations

Although LDI assesses blood flow with both spatial and temporal resolution, changes over time are captured with lesser precision when each perfusion cell is considered. This is because LDI measures blood flow in a single unit before moving on to the adjacent unit and so on to lastly put together a map of blood flow. Temporal resolution is achieved by repeating this procedure once one map is completed. However, as LDI measures only one unit at a time, measurement in each single unit is undertaken discontinuously, contrasting LDF, where continuous assessment is performed in a single point [34]. Therefore, rapid changes in skin blood flow are more difficult to assess over large skin areas using LDI than in single points using LDF. Multi-channel laser Doppler techniques were introduced to address this issue. Additionally, movement of the scanned area must be avoided since this will introduce recording artefacts. The necessity of remaining still might cause some discomfort to the subject. Technical dependence from environmental factors such as light, temperature, hairs, and humidity limits its application. Lastly, the technique lacks a standardized and widely accepted protocol [35].

### Perspective

Previous investigation indicated potential usefulness of LDI in research studies. Additionally, studies have reported that diabetic autonomic neuropathy can be verified early with simple LDI test in both type 2 and type 1 diabetes mellitus, highlighting the potential clinical usefulness of the technique [9, 23]. Research displayed reproducibility of axon-reflex-mediated vasodilation in repeated assessment via LDI indicating that the technique might be a reliable tool in longitudinal monitoring of vasomotor integrity. Whether this translates into clinical applicability remains to be answered. Moreover, research indicated the potential utility of LDI in the assessment of patients with neurodegenerative disorders. In patients with Alzheimer’s disease, LDI showed impairment of vasomotor function consistent with pathophysiological involvement beyond the central nervous system [36]. Diagnostic value of LDI in the clinical assessment of neuropathies remains to be determined.

## Laser speckle contrast imaging

### Procedure

When coherent monochromatic laser light hits a matt surface, the backscattered light generates an interference pattern on the detector. This high contrast grainy pattern is referred to as speckle pattern (of light and dark areas) [37]. Moving particles such as blood cells make the speckle pattern fluctuate, and the contrast decreases as the velocity of the caterers increases [38]. Similarly to LDI, laser speckle contrast imaging (LSCI) allows the assessment of skin perfusion over wide areas (up to 100 cm^2^) but, contrasting LDI, with a high frequency (up to 100 images/s). Computer simulations and in vitro measurements have shown that LSCI provides a perfusion index proportional to the concentration and mean velocity of red blood cells [39]. LSCI has high temporal and spatial resolution [38]. The skin penetration depth (300 mm) and the sensitivity to movement make up the differences between LSCI and LDI [40].

Local transient increase in cutaneous blood flow can be observed after various manoeuvers. Among those, local thermal hyperemia (LTH) allows evaluation of axon-reflex-mediated vasodilatory response, as opposed to post-occlusive reactive hyperaemia (PORH) which is mediated mechanically. In LTH, local heating of the skin induces local thermal hyperemia characterized by a biphasic rise in skin blood flow. A rapid initial peak is observed within 2–3 min after onset of heating, which mostly depends on a local sensory nerve axon-reflex [41, 42].

### Limitations

Skin penetration depth has been shown to be deeper for LDI (1–1.5 mm) than for LSCI (300 μm). However, penetration depth is highly wavelength-dependent: lower wavelengths measure the perfusion of capillary loops and upper plexus, whereas higher wavelengths allow measurement of deeper dermal circulation [43]. This should be considered because of the high variability of cutaneous thickness (e.g., epidermal thickness ranges from 60 mm on the eyelids to 800 mm on the palms of the hands). A direct comparison between LSCI and LDI has shown correlation over a wide range of human skin perfusion rates, despite a loss of correlation for low skin perfusion, suggesting a non-linear relationship between LSCI signal and skin blood flow [44, 45]. Also, LSCI is more sensitive to movement artefacts than observed with laser Doppler techniques. Commercially available devices provide arbitrary perfusion units (PU; 1 PU = 10 mV for laser Doppler). The relationship between PU and contrast is complex and requires computer processing of images obtained using LSCI [40].

### Perspective

Overall, the reproducibility of skin perfusion changes is higher when blood flow is recorded with LSCI compared to laser Doppler-based techniques, regardless of how data is expressed [46]. Clinical diagnostic utility of the technique remains to be determined.

Table 2 gives an overview of available tests of vasomotor function, their reproducibility, implementation, and underlying physiological pathways.

**Table 2:** Practical value and implications of techniques to assess vasomotor function

	Laser Doppler flowmetry (LDF)	Laser Doppler imaging (LDI)	Laser speckle contrast imaging
Arterial occlusion (PORH)	Sufficient validity Fair to poor reproducibility (improved when normalizing for skin temperature)	Slow kinetics with conventional LDI (can be improved by using multi-channel laser Doppler line)	Sufficient validity Easy to implement Very good reproducibility
Pressure (PIV)	Sufficient validity Laborious to implement (requires custom-made devices) Lack of data on reproducibility	Laborious to implement (requires custom-made devices) Lack of data on validity Lack of data on reproducibility	Laborious to implement (requires custom-made devices) Lack of data on validity Lack of data on reproducibility
Thermal local heating (LTH)	Sufficient validity Easy to implement Fair to poor reproducibility (improved when normalizing for skin temperature/ using integrated probes)	Laborious to implement Very good reproducibility	Laborious to implement Very good reproducibility
Local cooling	Sufficient validity Laborious to implement (requires custom-made devices) Acceptable reproducibility	Laborious to implement (requires custom-made devices) Lack of data on validity Lack of data on reproducibility	Laborious to implement (requires custom-made devices) Lack of data on validity Lack of data on reproducibility
Electric current (CIV)	Lack of data on validity Easy to implement Lack of data on reproducibility	Easy to implement Lack of data on validity Lack of data on reproducibility	Easy to implement Lack of data on validity Lack of data on reproducibility
Ach iontophoresis	Lack of standardized settings Easy to implement Fair reproducibility	Lack of standardized settings Easy to implement	Easy to implement Lack of data on validity Lack of data on reproducibility
Nitropusside iontophoresis	Lack of standardized settings Easy to implement Poor reproducibility	Easy to implement Lack of standardized settings Fair reproducibility	Easy to implement Lack of data on validity Lack of data on reproducibility
Time–frequency analysis	Easy to perform Heterogeneity in methods Lack of data on reproducibility	Difficult to perform	Difficult to perform

A table of techniques to assess vasomotor function sorted by assessment methodology and stimulus to induce blood flow response. Techniques are sorted by technology used to capture blood flow (column headings) and stimuli used to evoke blood flow response (left-sided headings)

## Assessment of sudomotor function

The integrity of small sudomotor nerve fibers can be assessed using axon-reflex-based diagnostic tests, of which the most widely used are described in detail below.

## Quantitative sudomotor axon reflex sweat test

The quantitative sudomotor axon reflex sweat test (QSART) is considered by many as today’s gold standard for evaluation of postganglionic sudomotor function. Philipp Low and colleagues developed the technique in 1983.

### Procedure

The sudomotor axon-reflex can be induced by Ach. Ach solution (usually 10%) is delivered into the upper dermal skin layers via iontophoresis evoking a local sweat response in the area of the delivery capsule (direct area). Efferent sudomotor nerve fibers are activated by binding of Ach to nicotinic and muscarinic receptors located at terminal nerve endings of efferent sudomotor nerve fibers. A local sweat response of the stimulated skin area within the capsule is induced (direct area). Additionally, an action potential within the sudomotor small fibers is generated. This potential travels antidromically to reach an axonal branching point, where it is redirected orthodromically to neighboring efferent sudomotor nerve fibers (indirect area). The evoked local sweat reaction is measured within the skin capsule as change of relative humidity, enabling an analyzation of temporal resolution, latency, magnitude, and duration of the tested subject’s sudomotor response. The sweat response is digitally analyzed and compared to established clinical standard values [47, 48]. The onset delay of the evoked sweat response after stimulation is gender-independent starting 1–2 min after iontophoresis of Ach in healthy subjects. The maximum sweat response is estimated approximately 5 min after the end of iontophoresis. The mean sweat output in contrast is gender-dependent and additionally depends on the tested body site. Normative values of mean sweat output for healthy female subjects are 0.25–1.2 μl/cm^2^ and for male subjects 2–3 μl/cm^2^ [47]. Tested body areas are commonly the forearms and the proximal and distal leg as well as the dorsal aspect of the feet.

### Limitations

QSART is the current gold standard of sudomotor function assessment and well-studied, but due to high technical demands, the technique is predominantly used in specialized centers for autonomic neurology. The conducting clinical staff needs to be trained adequately to guarantee a standardized clinical testing performance. Also, patients may experience a burning skin sensation or irritation due to iontophoresis, which is a general limitation of all axon-reflex-based test methods.

### Perspective

Broader clinical use of QSART to reliably diagnose patients with sudomotor dysfunction is under current scientific and clinical evaluation. Strategies to develop more user-friendly electrodes as well as Ach releasing gels to avoid leaking have been introduced [49].

## Quantitative direct and indirect test of sudomotor function

Quantitative direct and indirect test of sudomotor function (QDIRT) has been developed to establish an axon-reflex-based test of postganglionic sudomotor function with comparatively low technical demands [50].

### Procedure

Epidermal iontophoresis of 10% Ach solution is used to stimulate terminal endings of sudomotor small nerve fibers. Am action potential is generated and conducted to collateral sweat glands via the axon-reflex causing the indirect area of sweat reaction. To quantify the evoked sweat response and therefore sudomotor function, the tested skin region is covered with an indicator dye to visualize the occurrence of sweat droplets by color change of the indicator dye thereby visualizing sweat response. The color change is assessed by repeated digital photography with pictures taken every 15 s over 7 min. Based on the images, sudomotor function is digitally estimated with the aid of image analyzing software. The parameters of interest are number, size, and percentage of sweat droplets in the direct and indirect area of sweating over time [50]. Consequently, QDIRT allows a temporal and spatial analysis of the evoked sweat reaction.

### Limitations

Like QSART, QDIRT requires trained clinical staff in order to assess sudomotor function accurately to, e.g., avoid blurry pictures of the sweat response. One major limitation of QDIRT technique is that further sweat response in skin areas with already color-changed indicator dye will not be quantifiable. Moreover, the interindividual comparability of induced sweat response in QDIRT is relatively low due to not predefined skin areas [47]. Also, sweat droplets outside of the indirect area of sweat response might not be assessed due to missing indicator dye.

### Perspective

QDIRT needs yet to be evaluated concerning its capacity to detect pathological cutaneous small fiber dysfunction. A multicenter study is currently under way which comprises longitudinal use of QDIRT in patients with early stage Parkinson’s disease [51].

## Silicone impression mold technique

The silicone impression mold technique offers an axon-reflex test of sudomotor function which has lesser economical demands than well-established techniques such as QSART.

### Procedure

Like QSART, the silicone impression mold technique utilizes induction of an axon-reflex-mediated spread of sweating following iontophoretic application of a cholinergic agonist to assess functional integrity of sudomotor small nerve fibers. The silicone impression is applied for 5 min. It is then removed and evaluated for number and size of sweat droplets. The silicone impression mold technique is technically less demanding than other axon-reflex-based tests of sudomotor function such as QSART. A reduction in the number of sweat droplet impressions indicates dysfunction of sudomotor small fibers which is seen in various autonomic neuropathies such as diabetic neuropathy, hereditary sensory, and autonomic neuropathy or Fabry’s disease [52].

### Limitations

It is noteworthy that cutaneous artefacts such as debris and hair follicles may impact the impressions. Moreover, quantification of droplet impressions is rather time consuming as each silicone unit requires post hoc processing. Lastly, the silicone impression mold technique does not allow evaluation of the sudomotor axon-reflex response with temporal resolution [50]. A limitation of axon-reflex-based tests of sudomotor function is that a reduction in sweat response is not necessarily specific for dysfunction of postganglionic sudomotor small fibers, as impairment of eccrine sweat glands as well as damage or occlusion of their excretory ducts may also reduce the droplet count. However, separate analysis of direct sweating in the area of iontophoresis and indirect sweating in the axon-reflex region using the silicone impression mold technique or QDIRT may improve diagnostic discrimination between neurogenic and gland-related impairment.

### Perspective

This silicone impression mold technique provides the ability to assess the quantity of sweat droplets produced from individual sweat glands and is relatively inexpensive. However, widespread use of the technique as a clinical screening tool of small fiber neuropathy would require substantial standardization of the technique.

The silicone material would need to be non-occlusive, hydrophobic, and display fast polymerization characteristics. Moreover, preparation of the testing area on the skin as well as controlling for environmental factors are challenging in this regard.

## Non-axon-reflex-based sudomotor tests

A variety of tests of sudomotor function is available and most of them do not measure axon-reflex responses. The sympathetic skin response (SSR) assesses changes in skin conduction levels following sympathetic stimuli such as deep inspiration. The SSR is a well-established technique which is used in lie detection systems as it is very sensitive to sudomotor responses to emotional stimuli. However, the technique is not widely used as a clinical test as it is limited by high intra-individual and inter-individual variability [53]. The thermoregulatory sweat test (TST) utilizes controlled heating of the body to induce sweating. Using an indicator dye applied to the ventral body surface allows topographic analysis of sweating [54]. The TST can be combined with axon-reflex tests of postganglionic sudomotor function to differentiate postganglionic from preganglionic lesions. Preganglionic damage is characterized by an abnormal TST and normal QSART, silicone imprints, and QDIRT [47]. Up to now, the TST is provided only by highly specialized centers, due to the high technical demands. In fact, a fully equipped TST chamber is available in just a handful of autonomic testing centers. Sudoscan assesses electrochemical skin conductance. The technique uses reverse iontophoresis and chronoamperometry to assess the chloride ion concentration as a measure of sweating [55]. Although Sudoscan is easy-to-perform and inexpensive, the technique is limited by the interpretability of results. To date, it remains unclear whether Sudoscan stimulates sudomotor small fibers or the sweat glands directly (or both) [56]. The sensitive sweat test (SST) has been designed to assess secretion of each individual sweat gland along with their location, number, and distribution. Pilocarpin is applied using iontophoresis to induce a direct sweat gland-mediated response. Neurogenic responses have not been described. The SST allows for detailed assessment of sweat gland integrity and might complement primarily neurogenic techniques such as QSART in the clinical evaluation of sudomotor dysfunction [57]. The spoon test qualitatively evaluates the smoothness with which the convex side of a spoon slides over moist skin. It does not require any pharmacological stimulation of the sweat glands and is extremely easy-to-perform [58]. Its major limitation is the lack of quantitative assessment as the results depend on the investigator’s perception of the spoon sliding. Its major strength is the extremely easy and feasible testing protocol highlighting the technique’s potential as a clinical screening tool of sudomotor dysfunction.

## Assessment of pilomotor function

Pilomotor function has been less extensively studied compared to vasomotor and sudomotor techniques. To date, only a few papers on pilomotor function assessment have been published.

## Quantitative pilomotor axon-reflex test

The pilomotor axon-reflex test (QPART) stimulates noradrenergic fibers which sets it apart from available cholinergic-based vasomotor and sudomotor procedures. The direct peripheral stimulation of the pilomotor axon-reflex can also be evoked by mechanical, thermal, electrical, or pharmacological stimuli. Like sudomotor and vasomotor axon-reflex tests, iontophoretic stimulation of the axon-reflex appears to be a valid technique which in case of pilomotor testing differs from sudomotor and vasomotor tests by the choice of agonist applied to the skin. While sudomotor tests and most vasomotor tests utilize acetylcholine, QPART is performed using phenylephrine.

### Procedure

Iontophoresis of phenylephrine induces local piloerection in the area of phenylephrine application (direct region) and axon-reflex–response region (indirect region). Phenylephrine depolarizes the terminal nerve endings of cutaneous unmyelinated C-fibers (efferent pilomotor nerve fibers), which then evokes an action potential. Afterwards, the generated action potential is antidromically conducted to an axon branch point, where the action potential is transferred to neighboring axons and then orthodromically conducted to neighboring nerve terminals of C-fibers innervating the Musculi arrectores pilorum. Consequently, macroscopically visible goose bumps occur on the skin surface surrounding the iontophoresis skin region.

**Fig. 2:**
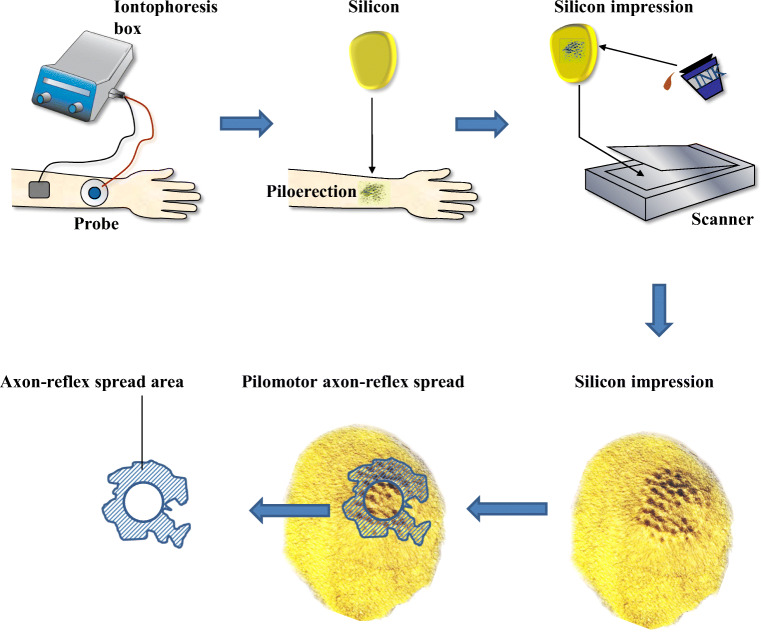
Quantitative pilomotor axon-reflex test. After stimulation with phenylepinephrine silicone, imprints of the tested skin area are taken and dyed afterwards. Digital analyzation is performed. The imprints are then analyzed for axon-reflex spread

The outline of the total area of piloerection is defined as the line connecting the edges of the most peripheral erect hair follicle impressions. The indirect region is calculated by subtracting the area of phenylephrine application from the total area of piloerection. Silicone impressions are used to create a local topographic map of piloerection. Local application of toner is used to increase the visibility of goose bump impressions and therefore allows image software-based digital analysis of pilomotor function. Silicone impressions of erect hair follicles can be quantified by the number of hair follicle muscle imprints in the areal extent of the axon-reflex response and the average volume of hair follicle muscular imprints in the indirect area (Fig. 2) [17].

In the process of developing the technique, a study in healthy subjects indicated that piloerection can be evoked by phenylephrine iontophoresis directly beneath the stimulation site and with latency in surrounding axon reflex regions. There, the response was shown to be of neurogenic origin. This was inferred from the observation that piloerection in the surrounding region is significantly reduced after pre-treatment with lidocaine and was abolished by lidocaine injection [17].

### Limitations

To date, QPART has been tested only in small populations of healthy subjects and patients with Parkinson’s disease and normative datasets from larger populations to compare individual patient data are lacking [9, 59]. Strong emotions and lowering of ambient temperature are common central provocative stimuli for piloerection which might be difficult to control for, particularly in the clinical setting.

### Perspective

Research has shown that pilomotor nerve fiber density is decreased in skin biopsies of diabetic subjects and subjects treated with topical capsaicin [16, 59]. Furthermore, research showed that pilomotor fibers are targeted by damage through alpha-synuclein deposition in the prodromal and early stages of synucleinopathies such as Parkinson’s disease [5, 11].

Viewed in conjunction with the aforementioned preliminary findings of pilomotor dysfunction in patients with Parkinson’s disease QPART, it might be speculated that QPART can be used to monitor disease progression as a surrogate marker of progressive structural small fiber damage. A longitudinal multicenter study is currently under way to test this hypothesis [51]. Additional potential applications of QPART include interventional studies assessing disease-modifying approaches.

## Conclusion

The established axon-reflex-based small fiber test of sudomotor, vasomotor, and pilomotor function allows specific assessment of postganglionic function. However, most of these techniques are limited by technically demanding settings and substantial interindividual variability. Therefore, comprehensive axon-reflex assessment is currently only available at specialized autonomic laboratories. Recent advancements include quantification of axon responses with both temporal and spatial resolution, e.g., via laser Doppler imaging, quantitative direct and indirect tests of sudomotor function or quantitative pilomotor axon-reflex test. The usefulness of this new generation of axon-reflex tests in the clinical assessment of neuropathy has yet to be determined.
